# Viability and Management Targets of Mediterranean Demersal Fisheries: The Case of the Aegean Sea

**DOI:** 10.1371/journal.pone.0168694

**Published:** 2016-12-29

**Authors:** George Tserpes, Nikolaos Nikolioudakis, Christos Maravelias, Natacha Carvalho, Gorka Merino

**Affiliations:** 1 Hellenic Centre for Marine Research, Institute of Marine Biological Resources and Inland Waters, Heraklion, Greece; 2 Hellenic Centre for Marine Research, Institute of Marine Biological Resources and Inland Waters, Athens, Greece; 3 Joint Research Centre, European Commission, Ispra, Varese, Italy; 4 AZTI-Tecnalia, Pasaia, Gipuzkoa, Spain; Aristotle University of Thessaloniki, GREECE

## Abstract

Management of the Mediterranean demersal stocks has proven challenging mainly due to the multi-species character of the fisheries. In the present work, we focus on the multi-species demersal fisheries of the Aegean Sea (eastern Mediterranean) aiming to study the effects of different management measures on the main commercial stocks, as well as to explore the economic viability of the fisheries depending upon these resources, by means of simulated projections. Utilizing the limited available data, our results demonstrated that, under the current exploitation pattern, the economic viability of the fleets is threatened, particularly if fuel prices increase. Additionally, the biological targets set for the most exploited species, such as hake, will not be met under the current management regime. The projections also showed that the only management scenario under which both resource sustainability and economic viability of the fisheries are ensured is the decrease of fleet capacity in terms of vessel numbers. In this case, however, measures to support the fisheries-dependent communities need to be implemented to prevent the collapse of local economies due to employment decrease. Scenarios assuming selectivity improvements would be also beneficial for the stocks but they showed low economic performance and their application would threaten the viability of the fleets, particularly that of the trawlers.

## Introduction

Mediterranean demersal fisheries are characterised by high diversity, both in terms of catch composition and the structure of the sector. The fisheries are typically multi-species, with a large variety of fishing gears being employed to exploit the stocks. Traditionally, most fishing activity takes place along the continental shelf and although catches are composed of more than 100 commercial species, their bulk comprise 5 to10 species, including hake, poor cod, red mullets, and shrimps [1, 2].

Generally, fishing vessels exploiting the demersal stocks can be classified in two major fleet categories: (a) bottom trawlers and (b) artisanal or small-scale coastal vessels. The artisanal fleet generally exploits the continental shelf using a wide variety of fishing gears (multi-gear fleet), mainly different types of static nets and bottom longlines. The artisanal fleet is typically composed of small sized vessels (<12m) that represent more than 80% of the total EU Mediterranean fleet. The bottom trawler fleet comprises larger-sized vessels accounting for about 10% of the total fleet [3].

Assessment of the Mediterranean demersal stocks falls under the responsibility of the General Fisheries Council for the Mediterranean (GFCM), which for management purposes has divided the Mediterranean Sea into 30 geographical sub-areas (GSAs). Until recently, due to the lack of systematic data collection in the Mediterranean, formal stock assessment reports were lacking or had a high degree of uncertainty for most stocks in the majority of the GSAs. In the last years, however, after the establishment of the EU Data Collection Regulation Framework (DCR and subsequent DCF) in the EU countries, and with the support of the Scientific, Technical and Economic Committee for Fisheries (STECF), formal assessments for the main commercial stocks in several European GSAs have been realized. Assessment results have shown that most stocks are over-exploited with reference to maximum sustainable yield (MSY) target reference points [4, 5].

In the Mediterranean, management of demersal stocks is exclusively based on various effort control regimes (input control). Direct regulation of effort is achieved through a licensing system allowing fishing in certain areas and seasons for specific vessels and gears, as well as through restrictions on the fishing capacity of licensed vessels (vessel tonnage, engine power). Direct effort regulation is typically accompanied by methods of indirect effort control, including various technical measures and management actions. Examples of such methods are closed areas and seasons, gear restrictions and minimum landing size regulations. In EU countries, apart from the management measures established at the national level, a series of common input control measures have been imposed through the Common Fisheries Policy (CFP) for the Mediterranean. Management targets follow the objectives of the CFP, which demands that the exploited stocks should meet MSY levels by 2020.

In the present work we focus on the demersal fisheries in the Aegean Sea (Eastern Mediterranean, GSA 22) aiming to examine the effects of different management measures on the main commercial stocks, as well as, to explore the viability of the fisheries depending upon these resources. These stocks are mostly exploited by the Greek fishing fleets and similarly to other Mediterranean areas, they are targeted by bottom trawlers and numerous artisanal vessels using a variety of fishing gears. The current state of the stocks is poorly known because assessments have not been performed in the last five years due to lack of relevant data.

A recently imposed management plan for the Aegean Sea bottom trawl fisheries is based on the results of surplus production modelling assessments that used landing statistics up to 2009 and a limited time series of survey data [6]. These assessments indicated that the hake stock was undergoing overexploitation, and stock decreases were to be expected if measures were not taken. Other important stocks, such as those of red and striped mullets were found to be exploited sustainably. The management plan includes temporal fishery closures, which, according to simulated projections, can drive the hake stock to MSY levels in line with the CFP objectives. Due to lack of analytical stock assessments, projections were based on logistic population growth models that inherently ignore the age structure of the stocks, as well as, important biological relationships (e.g. stock-recruitment) and changes in the fishery exploitation patterns. This implies that estimates are relatively uncertain. In addition, the delayed enforcement (beginning of 2014) of the management plan may have affected the expectations about stock increases.

Contrary to the approach used for the establishment of the existing management plan, in the present work we employ a more analytical approach to evaluate the effects of the applied management plan on important target stocks. In addition, we analyse economic data to consider the economic viability of the different operating fleets and explore alternative management measures in comparison to the status quo. Our approach uses the latest available fisheries data for obtaining analytical stock estimates and, based on these, attempts to forecast the medium-long term bio-economic effects of different management measures assuming uncertainty in various biological and economic parameters.

## Materials and Methods

**Fig 1:**
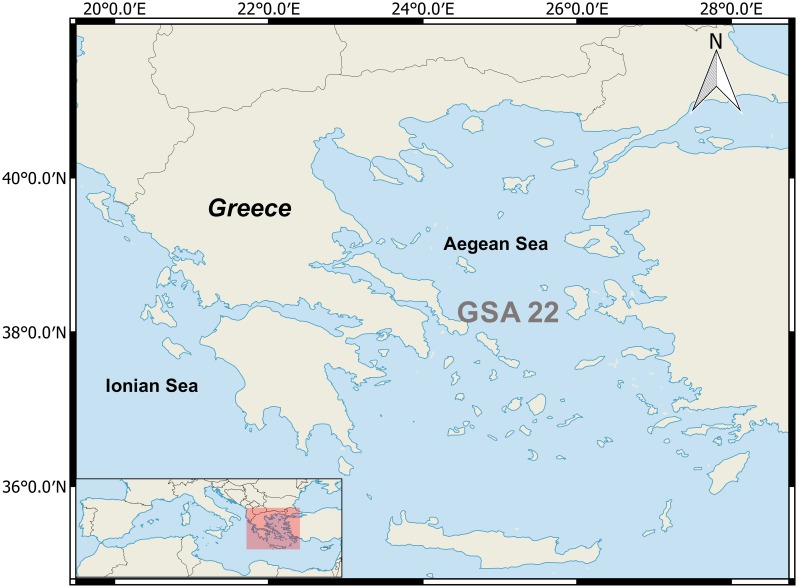
Map of the study-area.

The management strategy evaluation (MSE) approach described below considers, in an analytical way, the stocks of four demersal species in the Aegean Sea (Fig 1): hake (*Merluccius merluccius*), red mullet (*Mullus barbatus*), striped red mullet (*Mullus surmuletus*) and pink shrimp (*Parapenaeus longirostris*). According to the records of the Hellenic Statistical Authority these species represent an important fraction of the demersal trawl catches, their total catch fluctuating from 34–38% in the last decade. The mean contribution of each species in this period was 13%, 6.4%, 2.4% and 13.9%, for hake, red mullet, stripped red mullet and pink shrimp respectively. However, their contribution in terms of economic value is expected to be higher, given that they are among the most highly priced species [7]. They are also those closely monitored through the existing management plan for bottom trawlers. All stocks, excluding that of pink shrimp are exploited by both trawlers and artisanal vessels. Pink shrimp is captured solely by trawlers. The management scenarios were simulated using the FLR framework (the Fisheries Library in R, [8]) employing an operating model that consisted of three components: the population, fleet and observation models (R code in S1 File).

### Population model

The lack of a continuous series of fisheries data, did not allow the use of analytical age-based methods for estimating stock parameters. Thus, abundance and fishing mortality at-age for the examined stocks were obtained from cohort-analysis [9] assuming equilibrium conditions (pseudo-cohorts). Calculations were based on the mean catch-at-age data for the years 2006 and 2008 that were the latest available, given that collection of fisheries data through the EU co-funded Data Collection Framework (DCF) programme was not realised in the region in the years 2007, 2009–2013 and 2015, while it was only partially carried out in 2014. The estimated stock parameters are shown in Table 1.

**Table 1:** Abundance, fishing mortality, proportion mature and weight estimates at-age by species.

Species	Age	Abundance (N)	F_tr_	F_c_	Proportion mature	Weight (kg)
Hake	0	81279515	0.18	0.17	0.1	0.018
	1	38454913	0.44	0.31	0.6	0.063
	2	12107742	0.32	0.69	0.85	0.203
	3	2940852	0.17	0.65	0.95	0.44
	4	865422	0.01	0.43	1	0.752
	5	374704	0	0.43	1	1.92
Red mullet	0	193548560	0.61	0.11	0.17	0.014
	1	52049041	0.84	0.29	0.72	0.03
	2	9294619	0.98	0.31	0.89	0.05
	3	1407530	0.99	0.02	0.98	0.075
	4	279660	0.71	0.02	1	0.103
	5	74518	0.67	0.03	1	0.133
Striped red mullet	0	154728307	0.38	0.28	0.17	0.016
	1	43929455	0.21	0.74	0.72	0.035
	2	9295184	0.11	1.11	0.89	0.059
	3	1496492	0.09	1.07	0.98	0.088
	4	258178	0.06	0.91	1	0.125
	5	53542	0	0.9	1	0.179
Pink shrimp	0	745848037	0.61	0	0.18	0.003
	1	181485273	0.98	0	0.76	0.013
	2	30626898	0.95	0	0.9	0.021
	3	5307989	0.9	0	1	0.027

F_tr_ = partial fishing mortality of trawlers, F_c_ = partial fishing mortality of coastal vessels.

Given the lack of data series to properly estimate the parameters of a stock recruitment model, annual recruitment was derived from an empirical Beverton-Holt stock-recruitment (BH) relationship which included steepness. Following previous approaches of authors working with Mediterranean demersal stocks [5], steepness values were taken from a reverse Weibull distribution with h_mean_ = 0.74 and range between 0.2 (indicating a linear stock-recruitment relationship) and 1 (indicating SSB-independent recruitment). Estimates of asymptotic Spawning Stock Biomass (SSB) levels were obtained from pseudo-cohort analyses, by multiplying the derived abundance-at-age estimates by the proportion mature and by weight vectors shown on Table 1 (unpublished data) while assuming various levels of exploitation. In the case of hake, it was assumed that the estimated SSB was 30% lower than that corresponding to the maximum sustainable yield (SSB_msy_), while for the other three stocks it was assumed that the estimated SSB levels were equal to 90% of SSB_msy_ (Cohorts in S1 File). The above assumptions are in line with the existing management plan, which considers the hake stock moderately overexploited and the rest of the stocks within safe biological levels [6].

For each stock, numbers at age and year (N_*α*,*y*_; α > age at recruitment, y: year) for the projection years were derived from the standard exponential decay function.

$$N_{a,y}=N_{a-1,y-1}e^{-\left(F_{a-1,y-1}+M_{a-1,y-1}\right)}\;,$$ (1)

where *F* is fisheries mortality and M is natural mortality. In line with the assumptions introduced in the management plan, natural mortality rates were considered to be constant over age and equal to 0.4, 0.6, 0.6 and 0.8 for hake, red mullet, striped red mullet, and pink shrimp, respectively.

### Fleet model

Two fleet segments were considered: (a) the bottom trawlers (*TR*) and (b) the polyvalent coastal vessels (artisanal or small-scale fleet) that use various types of static nets and longlines (*CO*).

For each fleet segment:

Fishing mortality by age and year (*F*_*α*,*y*_) were determined by a separable model:

$$F_{a,y}=S_{a}F_{y}\;,$$ (2)

where *S*_*α*_ denotes the selection at age.

The relationship between *F*_*y*_ and fishing effort (*f*_*y*_), expressed in terms of days at sea was modelled as:

$$F_{y}=q_{y}f_{y}\;,$$ (3)

where *q*_*y*_ is the catchability considered constant over time.

Hence, eq (2) becomes:

$${\text{F}}_{\text{a},\text{y}}={\text{S}}_{\text{a}}{\text{q}}_{\text{y}}{\text{f}}_{\text{y}}$$ (4)

Annual catches by age and year (*C*_*α*,*y*_) were generated using the standard Baranov equation:

$$C_{a,y}=\frac{F_{a,y}}{Z_{a,y}}N_{a,y}\left(1-e^{-\left(F_{a,y}+M_{a,y}\right)}\right)$$ (5)

The annual value of landings (gross revenue, *GR*_*y*_) for the examined species was estimated from:

$$GR_{y}=\sum_{a}\left(C_{a,y}P_{a,y}\right)\;,$$ (6)

where *P*_*α*_ denotes the price per kilo at age.

Based on interviews with fishers, the catch volume of the rest species was estimated empirically, as a linear function of either the red mullet or hake catches for trawlers and coastal vessels, respectively. Total variable (operational) costs by year (*VC*_*y*_) were estimated from:

$$VC_{y}=\bar{D_{y}}f_{y}\;,$$ (7)

where $\bar{Dy}$ denotes the mean daily operational costs. Total fixed costs by year (*FC*_*y*_) were estimated from:

$$FC_{y}=\bar{V_{y}}n_{y}\;,$$ (8)

where $\bar{Vy}$ is the mean annual fixed vessel costs and *n*_*y*_ the number of vessels.

Fish prices, as well as, fixed and operational costs were considered constant throughout the projection period and were based on the mean values estimated from interviews with fishers in 2013 (Table 2). The market value of ages corresponding to undersized catches was assumed zero. In the case of fuel costs (included in variable costs), however, two different rates were assumed in the MSE scenarios, based on the mean fuel prices of 2013 and 2015 respectively. Finally, net revenue (*NR*_*y*_) was estimated from:

$$NR_{y}=GR_{y}-\left(VC_{y}+FC_{y}\right)$$ (9)

**Table 2:** Initial conditions in economic parameters.

**Market prices/species in €**	**Age**	**Price/kg**
Hake	0	0
	1	6
	2	8
	3	10
	4	12
	5	13
Red mullet	0	0
	1	8
	2	10
	3	12
	4	17
	5	20
Striped red mullet	0	0
	1	9
	2	11
	3	14
	4	17
	5	23
Pink shrimp	0	0
	1	5
	2	7
	3	10
Other species (trawlers)	All ages	6
Other species (coastal)	All ages	11
**Costs per vessel in €**	**Trawlers**	**Coastal**
Annual Fixed costs	20400	3200
Mean daily variable costs	1355	109

### Observation model

The abundance-at-age estimates of the pseudo-cohort analysis were used to generate the initial populations assuming log-normal errors with a coefficient of variation (CV) equal to 20%. Log-normal errors with CV = 45% were assumed for the estimates of the stock-recruitment relationship and, based on interviews with fishers, CVs equal to 10 and 40% were assumed for the operational costs of the trawler and artisanal small-scale fleets, respectively. Hence, abundance, recruitment and operational cost rates were drawn randomly from the corresponding distributions.

### Scenarios

Four management scenarios were simulated 200 times for a period of fifteen years and estimates of SSB, catch and net revenue per vessel were obtained by year. As not any catch-at-age data were available after 2008, to allow for fishing mortality estimates, it was assumed that fishing mortality during the 2009–2014 period varied according to the fleet size changes recorded in the Greek Fleet Registry considering also the temporal closure implemented to the fisheries from 2014 onwards in the frame of the management plan. It should be noted that the size of the fishing fleet has been progressively reduced by about 15% during that period (Table 3). For each fleet component, the reference (base case) selectivity (*S*_*α*_) estimates were derived from the pseudo-cohort analysis:

$$S_{a}=\bar{\frac{F_{a}}{\bar{F}}}$$ (10)

**Table 3:** Evolution of the fleet size, in terms of numbers of vessels, from 2009–2014.

Year	Trawlers	Artisanals
2009	309	14918
2010	294	14866
2011	293	14394
2012	283	14047
2013	271	13610
2014	270	13590

The four scenarios examined were:

1. Status quo. A continuation of the currently implemented management plan was foreseen up until the final year of the simulation. The current management plan, implemented in 2014, defines that the trawl fishery is closed for 4.5 months per year, while coastal fisheries capturing hake are not allowed to fish in February (considered as peak spawning month for hake in the area). It was assumed that selectivity will be similar to the corresponding reference estimates for the entire projection period, while fishing effort was considered constant from 2014 onwards.
2. Decrease of fleets’ capacity, expressed in terms of number of vessels, by 20% from 2015 onwards. No change in the selection pattern was assumed, while fishing effort was assumed to decrease proportionally to the fleet size.
3. Fishery closure for an additional month for all fleet components implemented from 2015 onwards. No change in the selection pattern was assumed, while fishing effort was reduced accordingly.
4. Selectivity changes of the bottom trawl gear that would improve the current selection pattern for hake [10] resulting in negligible catches of undersized hake individuals (<20 cm). Considering the growth pattern of hake [11] a 95% selection decrease was assumed for age 0, while for red and striped mullets, given their smaller size at age [12], the same decrease for ages 0–2 was assumed. For pink shrimp a 95% selection decrease for age 0 was also assumed based on existing selectivity studies and the species growth pattern [13, 14]. This selection pattern was assumed constant from 2015 onwards.

## Results

**Fig 2:**
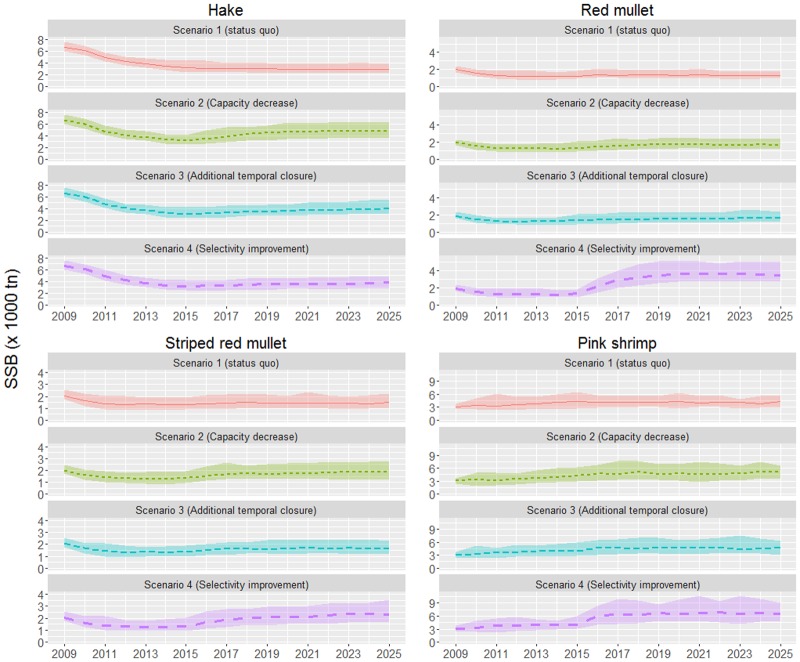
Spawning Stock Biomass by species (in 000’s tons) under the four simulated scenarios. Dotted lines indicate the median estimates and the upper and lower limits of each ribbon represent the 0.1 and 0.9 quantiles.

Projections demonstrated that the 2015 SSB levels for all species, except that of pink shrimp, were lower than those estimated at the beginning of the examined period (Fig 2). Particularly for hake, which is considered as the most vulnerable stock, SSB showed a steadily decreasing trend, while SSB for the three other species remained stable over the last five years (2011–2015).

Under the status quo scenario, the hake SSB will be stabilised around the 2015 levels and will be about half of the biomass estimated at the beginning of the period. For the other species, SSB at the end of the examined period will be about 10% higher than the 2015 rate. Scenario 2 simulating a 20% capacity reduction would clearly lead to significantly higher (~50%) hake SSB levels than scenario 1 (status quo), while scenarios 3 (fishery closure) and 4 (selectivity improvement) would result in moderate increases (~30%). Scenario 4 and, to a lesser extent, scenario 2 will be the most beneficial for the rest of the stocks. Implementation of scenario 4, in particular, would nearly double the SSB of red mullet and pink shrimp. Scenario 3 will produce intermediate SSB levels between the status quo and scenario 2 (Fig 2).

**Fig 3:**
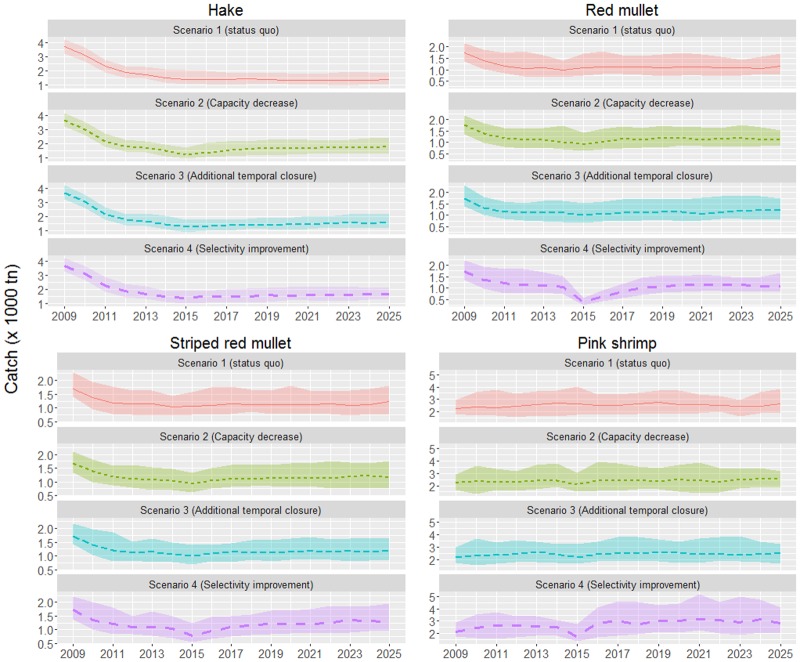
Catch by species (in 10^3^ tons) under the four simulated scenarios. Dotted lines indicate the median estimates and the upper and lower limits of each ribbon represent the 0.1 and 0.9 quantiles.

Differences among scenarios in the medium-term catches (including undersized individuals) are rather small and modest increases (up to 20%) in relation to status quo are expected for certain species, mainly from scenarios 2 and 4. In particular, scenario 2 will benefit hake and red mullet catches, while scenario 4 will favour the increase of striped red mullet and pink shrimp ones. In most cases, the selectivity improvement scenario will produce relatively lower catches for a transitional period of 1–3 years after its implementation (Fig 3).

**Fig 4:**
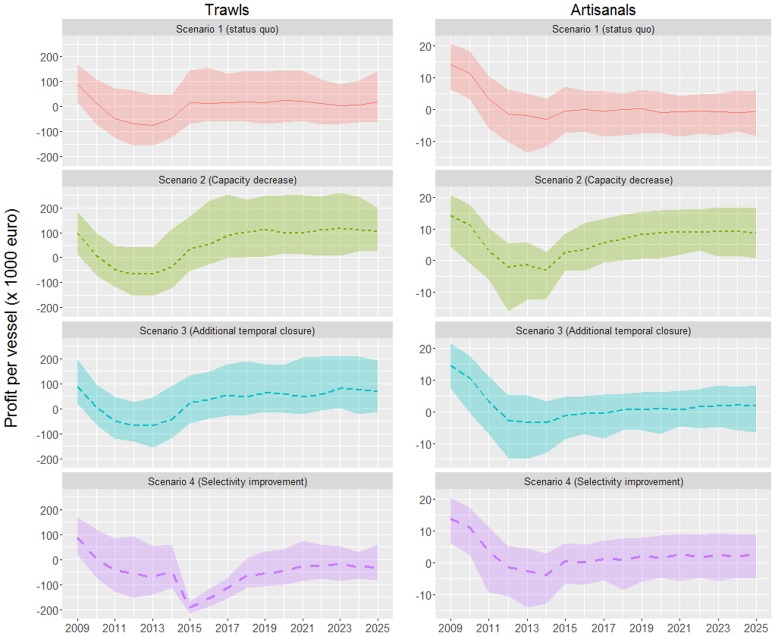
Profit per vessel (in 10^3^ €) for the trawl (left graph) and artisanal fleet (right graph) under the four simulated scenarios, assuming that fuel prices from 2016 onwards are equal to the 2015 mean rate. Lines indicate the median estimates and the upper and lower limits of each ribbon represent the 0.1 and 0.9 quantiles.

**Fig 5:**
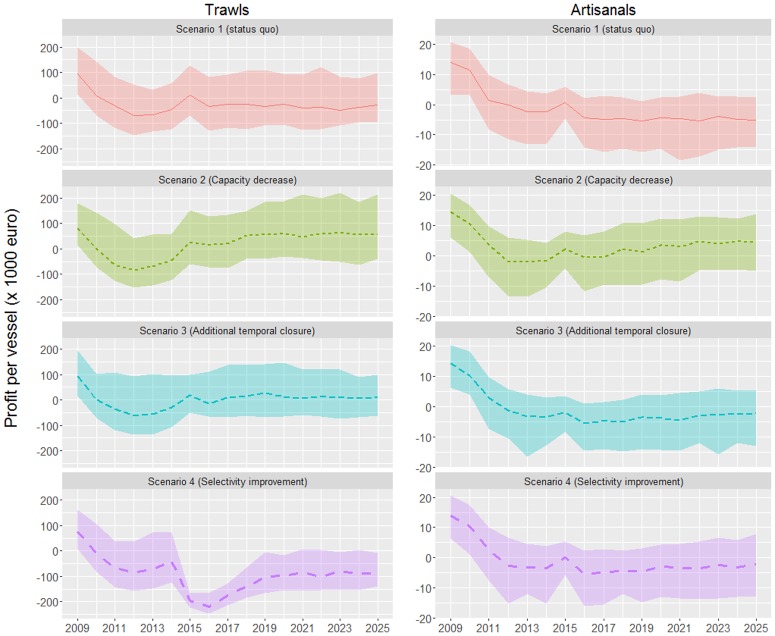
Profit per vessel (in 10^3^ €) for the trawl (left graph) and artisanal fleet (right graph) under the four simulated scenarios, assuming that fuel prices from 2016 onwards are equal to the 2013 mean rate. Lines indicate the median estimates and the upper and lower limits of each ribbon represent the 0.1 and 0.9 quantiles.

The capacity reduction scenario is by far the best one in terms of profits per vessel for both fleet segments and it is the only scenario securing medium term profits (the lower limits of the estimates are almost all above zero), independently of fuel prices (Figs 4 and 5). Under the assumption of low fuel prices (2015 levels), the fishery closure scenario is the second best for the trawlers, while for the artisanals it provides comparable profits with the selectivity improvement scenario. The latter is the worst for the trawlers and threatens their viability, particularly if fuel prices increase to 2013 levels. In that instance, the status quo is not sustainable for any of the fleet segments and while, apart from the capacity reduction, the fishery closure scenario seems to sustain profits for the trawlers, the only viable solution for the artisanal vessels is the capacity reduction (Fig 5). The status quo is also problematic for both fleet segments even under the assumption of low fuel prices (Fig 4).

## Discussion

Elaboration of management objectives in multi-species fisheries is a difficult challenge. The EU Common Fisheries Policy (CFP) considers achieving maximum biological sustainability by 2020 as the management target for the exploited fish stocks. In the case of the Mediterranean demersal stocks that are managed through input control schemes this implies that the exerted fishing pressure should correspond to fishing mortality rates that are able to sustain exploitation of the stocks at MSY levels. It is obvious, however, that in multi-species fisheries it is rather impossible to achieve MSY targets across all stocks simultaneously by any management measure. Hence, it seems more appropriate to focus on the sustainability of the most threatened stock(s), while monitoring the response of the less threatened stocks to the implemented management measures.

In line with this approach, we attempted to evaluate if the currently applied management measures in the Aegean Sea (status quo scenario) could achieve their main target, which is the adjustment of hake stock to MSY levels. Similarly to the Aegean Sea, hake stocks are considered to be overexploited in most Mediterranean regions, as revealed by the stock assessments carried out by the STECF [15]. It is very likely that the implementation of EC regulation 1967/2006, which bans bottom-trawl activities within 1.5 nautical mile off the coast, has resulted in the shifting of fishing activities towards deeper waters; thus increasing fishing pressure onto slope resources, such as those of hake [16].

Our results indicated that although hake biomass will stop decreasing, it will be stabilised at levels far lower than those observed in the beginning of the period (late 2000’s), which were considered to be the optimum ones [6]. As implied from Fig 2 the hake stock biomass kept decreasing up to 2014 and the recent effort reductions introduced through the implementation of the management plan in that year, seem to be able to stop this decreasing trend but not to support biomass re-establishment to desirable levels. Probably, if the management plan had been implemented a few years earlier, the expectation of hake biomass rebuilding to MSY levels might have been fulfilled. Under the current conditions, however, only the capacity reduction scenario would lead to substantial increases of hake SSB, while the selectivity scenario will be the most beneficial for the rest of the stocks. However, none of the scenarios will succeed in bringing hake SSB to the levels observed in the beginning of the period.

According to the profit per vessel projections, the viability of both fleet segments is threatened under the status quo scenario, particularly if fuel prices increase (Figs 4 and 5). Fuel prices have a crucial impact on fisheries, and in the case of trawlers, fuel costs represent about 40% of their total costs [17]. It should be noted that market prices do not seem to follow fluctuations in operational costs as they have remained rather stable over the last years even though fuel prices varied up to 40% (unpublished data). Based on this market stability, fish prices were assumed constant throughout the projection period in all examined scenarios. Another important aspect to take into account is that the income estimates are based on the assumption that undersized fish are not commercialized. In fact, this depends on the degree of implementation of the existing minimum size regulations. In cases that these are not fully implemented, some undersized fish may enter into the market chain, allowing for further increases of the model-estimated income of fishers.

The capacity reduction is by far the best scenario, especially for the artisanal vessels as it is the only one securing their viability under all conditions. (Figs 4 and 5). Under this scenario, although overall catches will not show any major increase, they will be distributed among fewer vessels, resulting in higher income per vessel. In addition, as a result of the decreased fishing pressure, catches will tend to include bigger fish that are generally higher priced. This will also happen under the selectivity improvement scenario, which will lead to an increase of the mean size of fish in the catch, as smaller individuals will not be available to the trawl gear. Regarding trawlers, although this scenario will increase pink shrimp catches (a species targeted solely by trawlers) by ~20% in relation to the status quo, this cannot counterbalance the economic losses ought to the decreased captures of smaller than 20cm fish, such as young individuals of red and striped mullets. This seems to be the main reason for the very low economic performance of the selectivity scenario in the case of trawlers. In addition, trawlers will not benefit much from the hake biomass increases expected from the selectivity scenario, as bigger hake is inhabiting deeper waters (>350m) that are hardly accessible to the trawl fishery [16].

It can be argued, that is preferable to interpret relative trends rather than absolute values from the current projections, given that the assumptions about stock-recruitment relationships have affected stock biomass, catch, and, consequently income estimates. However, the catch estimates of the examined species in 2014 obtained from the partial completion of the DCF in that year are within the limits of the projected values (HCMR, unpublished data). This suggests that the scenario parameterisation that was adopted in this study seems rather realistic. Additionally, current findings are in line with past studies in the Mediterranean, which have shown that the current exploitation rates of several stocks are not sustainable and have expressed concerns about the economic viability of the fisheries [4, 5, 18].

Previous studies focusing on the western and central Mediterranean regions have suggested that selectivity improvements would be much more effective in improving stock status than decreases in exploitation rates [4, 5]. Although findings of the different studies are not directly comparable, as different assumptions have been employed regarding selectivity, our results are in line with the above findings indicating that for all species except hake, the highest SSB increases are expected from selectivity improvements. In all cases, however, the lack of relevant data has not allowed simulation of actual gear modifications and all assumptions about selection patterns are rather intuitive based on expert knowledge. The current study in particular, is based on an age-based model; thus selectivity assumptions were approximated, given that all relevant studies and minimum size regulations refer to size.

In reality, improvements in selectivity demand the adoption of gears with larger mesh sizes than those currently used and such changes were already implemented a few years ago through EU Regulation 1967/2006. Given the multi-species nature of the fisheries and the, relatively, small size of several Mediterranean commercial fish species, further mesh-size increases would likely result in decreased catches of such species (e.g. picarel); thus it is difficult to be widely accepted and thus effectively implemented.

The current study suggests that management of the demersal resources would be more efficient through fleet capacity control schemes, as this will ensure the viability of all fleet segments. Although capacity assessments in the Mediterranean are limited to very few fisheries (e.g. [19, 20, 21], overcapacity is generally considered as one of the main drivers behind overfishing in the EU waters and partly responsible for the failure of the previous CFP [15]. Regarding the Greek demersal fleets, their capacity in terms of number of vessels has been reduced by 15% in the last 6–7 years but the Greek fleet is still the most numerous within the EU. Assuming no compensation in effort, the present results suggest that a further reduction up to 20% would be beneficial for the stocks, ensuring also the viability of the fisheries. It can be argued that the so called technological creep could counteract the capacity reduction as a relevant study conducted in the Aegean Sea estimated an annual technological creep increase of up to 0.79% for the period 1994–2008 [22]. However, given the lack of fleet modernisation programs in the last decade, it is rather difficult to justify such an increase in the last 5–8 years.

Relatively modest effort reductions, such as the one-month fishery closure tested in scenario 3, do not seem to provide major improvements in terms of stock biomass and economic profits. Samy-Kamal et al. [23], based on landings data from fisheries operating along the Alicante coast (south-western Mediterranean), concluded that a seasonal closure would have positive biological effects for some target species but this depends on its timing. Merino et al. [17], who studied the performance of demersal fisheries in the western Mediterranean, found that only drastic fishing effort reductions (48–71%) in terms of days at sea would improve the health of fish stocks and increase the economic profits of the bottom trawl fishery. Probably such drastic effort cuts would also be beneficial in the current case but they are rather unrealistic and difficult to be widely accepted, particularly for the Aegean bottom trawl fishery, which is already closed for 4.5 months per year. As it has been found in past studies, it is very likely that drastic fishery closures would seriously decrease catches and fisher’s income, at least for a transitional period, until stock size improvements [24].

Apart from the Greek fisheries considered in the present study, the demersal resources of the Aegean Sea are also exploited by other national fleets, mainly Turkish ones. Based on the latest FAO statistics, catches of those fleets are estimated to be about 1/5 of the Greek ones but further information that is necessary for analytical assessments (effort, size composition of the catch, etc.) is lacking. Therefore, in line with the STECF approach, our assessments were solely based on the Greek fisheries data. Certainly, this may have affected to some extent our estimates, probably resulting in more optimistic expectations from the examined scenarios, regarding SSB, catches and profits. However, it should not have any major impact on our conclusions regarding the relative performance differences among the examined management scenarios and their potential in supporting the sustainability of the demersal fisheries in the Aegean Sea. The main argument of the reformed CFP is that managing stocks according to MSY will mean going from fishing desperately on smaller fish stocks to fishing rationally on abundant ones (EC Reg 1380/2013). In the present study, the sustainability of the stocks and the viability of all fisheries is primarily achieved through fleet size reduction (20%) assuming no compensation in effort. Such a management measure, however, will affect employment, not only in the fisheries sector, and may have important social impacts. Thus, it should be carefully planned and subsidies accompanied by ad hoc programs aimed at creating alternative jobs may be necessary to support fisheries-dependent communities. To this end, relative provisions have already been foreseen in the European Maritime and Fisheries Fund (EMFF) (EC Reg 508/2014) and can be utilised to make such transitions easier for the fishing industry.

## Supporting Information

S1 File Contains Cohorts file and R code file for the reproduction of the analysis. (ZIP)
